# Emotional intelligence among medical students: a mixed methods study from Chennai, India

**DOI:** 10.1186/s12909-018-1213-3

**Published:** 2018-05-04

**Authors:** Subashini Sundararajan, Vijayaprasad Gopichandran

**Affiliations:** 1 ESIC Medical College & PGIMSR, KK Nagar, Chennai, 600078 India; 2 Department of Community Medicine, ESIC Medical College & PGIMSR, KK Nagar, Chennai, 600078 India

**Keywords:** Emotional intelligence, Medical curriculum, Doctor patient relationship, Cognitive reflection, Fish bowl discussion

## Abstract

**Background:**

Emotional Intelligence is the ability of a person to understand and respond to one’s own and others’ emotions and use this understanding to guide one’s thoughts and actions. To assess the level of emotional intelligence of medical students in a medical college in Chennai and to explore their understanding of the role of emotions in medical practice.

**Methods:**

A quantitative, cross sectional, questionnaire based, survey was conducted among 207 medical students in a college in Chennai, India using the Quick Emotional Intelligence Self Assessment Test and some hypothetical emotional clinical vignettes. This was followed by a qualitative moderated fish-bowl discussion to elicit the opinion of medical students on role of emotions in the practice of medicine.

**Results:**

The mean score of Emotional Intelligence was 107.58 (SD 16.44) out of a maximum possible score of 160. Students who went to government schools for high school education had greater emotional intelligence than students from private schools (*p* = 0.044) and women were more emotionally intelligent in their response to emotional vignettes than men (*p* = 0.056). The fish bowl discussion highlighted several positive and negative impacts of emotions in clinical care. The students concluded at the end of the discussion that emotions are inevitable in the practice of medicine and a good physician should know how to handle them.

**Conclusions:**

Medical students, both men and women, had good level of emotional intelligence in the college that was studied. Students from collectivist social settings like government high schools have better emotional intelligence, which may indicate that a collectivist, community oriented medical education can serve the same purpose. Though students have diverse opinions on the role of emotions in clinical care, cognitive reflection exercises can help them understand its importance.

**Electronic supplementary material:**

The online version of this article (10.1186/s12909-018-1213-3) contains supplementary material, which is available to authorized users.

## Background

Emotional intelligence may be defined as the ability of a person to understand and respond to one’s own and others’ emotions and use this ability to guide one’s thoughts and actions [1]. Emotional intelligence is essential for all human interactions. The ‘emic’ perspective of emotional intelligence helps a person understand and regulate their own emotions and use them for effective human interactions. The ‘etic’ perspective of EI helps them relate to the emotions, empathize and respond to the emotions of others. Both these perspectives are essential for effective human interactions [2].

In medicine, a profession that thrives on human interactions, emotional intelligence is of great importance [3]. There is increasing interest in the recent times on the importance of emotional intelligence for effective clinical practice. Empathy and compassion have always been desirable virtues in a doctor [4]. The ability to empathize improves clinical interactions as well as brings out good clinical outcomes [5]. Studies have shown that physicians who demonstrate empathy are more effective in eliciting a good history, arriving at an accurate diagnosis and eliciting good compliance to their prescribed treatment [6]. Empathy and compassion in the physician also makes the patients perceive a sense of well-being, which speeds their recovery [4–7]. These findings emphasize the importance of emotional intelligence in clinical care.

Emotional intelligence is not just important for providing good clinical care, it is also important for managing all the human relationships that happen as part of the medical treatment process. Emotional intelligence is important for the physician to effectively work as a team among nurses, hospital managers, and other allied health professionals [8, 9]. It is important to also effectively communicate with the relatives, friends and family of the patients under their treatment [10]. In recent times we are witnessing several instances of violence against doctors and health care professionals in India and many low and middle income countries [11, 12]. The vulnerability of the patients and their relatives, the uncertainty associated with the treatments, overcrowded hospitals and overworked health care providers contribute to many of these violent episodes. They can also be traced back to lack of emotional intelligence among the physicians and health care professionals, thus aggravating the situation and pushing the frustrated patients and their relatives to violence.

Therefore, there is a need to impart emotional intelligence skills as part of medical education in creating sensitive and empathetic physicians for the future. The medical curriculum is overloaded with subject content, that often there is very little time allotted for skill development. Much of these soft skills such as effective communication, emotional intelligence, empathy etc. are left to subconscious learning by observation of senior colleagues in action at the bedside and the outpatient clinics. In recent times, active teaching-learning methods are advocated for imparting empathy, emotional intelligence and communication skills in the medical curriculum in many countries. In India, the Vision 2015 document of the Medical Council of India, the apex body in charge of curriculum development for medical education in the country, focussed on the ATCOM module, which is the Attitude and Communication module, in order to impart education on communication skills, empathy, emotional intelligence and ethics [13]. However, implementation of this curriculum in the actual teaching-learning process leaves much to be desired. In this study, we attempted to assess levels of emotional intelligence among the students in a medical college in Chennai, India and to explore their understanding of the role of emotions in medical practice. We used a mixed methods assessment.

## Methods

Initially we conducted a quantitative questionnaire based survey to measure the emotional intelligence of the students and followed this up with a qualitative research method to gain a deeper understanding of the students’ attitudes towards the role of emotions in the practice of medicine.

### Study setting

Our institution is a tertiary care teaching hospital and medical college located in the heart of the city of Chennai in south India. The students are admitted to the college through three channels – firstly, from a merit list generated from the marks obtained in the school-leaving exam conducted by the State Government, secondly from a merit list generated from an eligibility test conducted at the national level and thirdly a dedicated quota for children of employees who have a low income and are insured under the social welfare health insurance scheme of the country. The medical education is highly subsidized by funding through the social welfare health insurance scheme. All the students are required to serve for a period of 5 years as medical officers for the social welfare health insurance scheme at the end of their medical course. Students in the college come from diverse backgrounds, rural, urban, middle socioeconomic class and some from even modest socio-economic backgrounds.

### Preliminary quantitative survey

We conducted the preliminary quantitative survey among students of the second, third and final year of medical school. The first year students were not included as they did not yet have exposure to clinical rotations. A total of 300 students which was the number enrolled across these years, were approached for the study.

We developed a survey instrument containing the following components: (1) Socio-demographic characteristics of the student including age, gender, nativity (urban/rural) and type of high school education (private/government) (2) Quick Emotional Intelligence Self Assessment Test (3) Four clinical vignettes of emotional situations and responses to these vignettes. We gave these survey questionnaires in a pen and paper format to the students during their class hours. They were requested to fill out the questionnaires and return it to us in confidence. We anonymized the questionnaires and took all measures to ensure strict confidentiality. The questionnaire used for the survey is provided as Additional file 1.

The Quick Emotional Intelligence Self Assessment Test comprises of four domains namely: a) Emotional Awareness, b) Emotional Management, c) Social Emotional Awareness and d) Relationship Management [14]. Participants’ response to each item was based on a 5-point frequency type of Likert scale ranging from never to always (0–4). Before conducting the survey we gave the scale to a clinical psychologist and a medical education expert and had the scale content validated for the local context.

The clinical vignettes were developed from the real life clinical practice of the second author. These vignettes captured four clinical scenarios which can be described as emotionally demanding, namely, angry patient, demand for irrational treatment, crying patient and lonely patient demanding traditional treatments. We developed the response options to these questions based on mutual consensus as well as discussion with other senior physicians. The most appropriate responses were identified using a Delphi technique discussion among physicians. Three physicians, one internist, and two general practitioners were invited for the Delphi discussion. Their responses were discussed over 4 rounds and the responses to these vignettes were arrived at through consensus. Each vignette had 4 responses and they were ranked and scored between 4 and 1 from the most emotionally intelligent response to the least. The clinical vignettes and responses were presented as multiple-choice single response format to the participants. Their responses were scored based on the ranking that was established.

Collected data were entered in Epi Info software version 7.2 and analysis was performed using SPSS Statistical software version 21.0. Simple frequencies and descriptive statistical analysis were performed and reported. The Chi Square statistical test of significance was applied and *P* ≤ 0.05 was taken as a statistically significant association. Multiple logistic regression analysis was performed to identify factors influencing EI.

### Follow up qualitative study

After we analysed the quantitative study findings, we got a broad understanding of the level of emotional intelligence of the medical students and their responses to the emotional clinical vignettes. Based on this understanding we wanted to explore the ideas that students had on importance of emotions in the practice of medicine and to further probe their emotional intelligence in a clinical context. To achieve this, we conducted a qualitative study using a fish-bowl discussion.

A fish bowl discussion was organized among students studying in the final year. In the final year, students have clinical rotations in internal medicine, surgery, obstetrics and gynaecology, paediatrics and orthopedics. A total of 86 students participated in the discussion comprising of 50 women and 36 men. The entire discussion was recorded and transcribed verbatim with verbal consent from the students.

The students were made to sit in a big circle. There was a smaller circle of 5 discussants within the larger outer circle. These 5 discussants started the discussion on role of emotions in the practice of medicine. As the central group of discussants continued the discussion, participants from outside the circle stood up and replaced one of the central discussants, when they felt that they had something more to add to that particular speaker or when they wanted to object to what was said by the speaker. This discussion was continued till saturation of the discussion points was achieved.

The second author acted as facilitator for the discussion. He used probes in the beginning to elicit the discussions. When discussions went off track, the facilitator intervened, summarized the discussions and used specific probe questions to bring the discussions back to track, such as:

- What are the common emotions encountered in medical practice?
- What is the impact of these emotions on medical decision making?
- Can these emotions be controlled and regulated?
- Can these emotions be completely removed?
- Are these emotions helpful in some situations?

The total discussion last for around 1 h and 10 min. There were totally 12 speakers who actively participated in the discussion with a total of 8 replacements of the central five discussants. Two speakers requested for re-entry into the central circle after being replaced and were allowed with the larger group consensus. It is the nature of a fishbowl discussion that the distance between the core speakers and the audience is reduced and the audience themselves sometimes become speakers. It is a communication technique where many of the audience feel engaged with the core discussion, even without participating. Thus, it is a very useful cognitive reflection activity. Though all the 86 participants did not actively contribute to the core discussion, they were engaged in the cognitive reflection.

The facilitator also kept track of the mood and tone of the discussion. At the end of the discussion, the facilitator summarized all the key points discussed from his notes. He also made his observations about the important changes in the mood of the group and dynamics of the discussion. All the participants were given a chance to agree or challenge the interpretations and reflect on the key points summarized.

The qualitative discussion data was analysed using a thematic analysis framework. The transcript of the fish bowl discussion was read in detail by the second author and open coding performed to label the concepts and ideas in the transcript. During the second round of analysis, the codes were grouped into emergent themes. These emergent themes were then described, and appropriate verbatim quotes identified to substantiate each theme. The coding was reviewed by a panel of experts comprising of social scientists, psychologists, clinicians, when the findings were shared in a workshop.

### Ethical considerations

The study was presented in the Ethical Committee of the institution of origin of this study and was approved after expedited review. Written informed consent was obtained from all participants who responded to the quantitative survey. There was no consent obtained from the participants of the fish-bowl discussion as it was part of a teaching-learning process. The Ethics Committee approved the informed consent waiver. Information about the fish-bowl discussion and use of the data obtained from it for scientific analysis was provided to the participants before the start of the discussion.

## Results

### Quantitative survey

Out of a total of 300 students approached for the study, 207 consented to participate and returned the filled questionnaires. The response rate of the study is 69%. The demographic characteristics of the participants have been depicted in Table 1.

**Table 1:** Characteristics of the study population

S.No	Characteristics	Category	Frequency (%)
1	Age (years)	18	4 (1.93%)
		19	36 (17.39%)
		20	80 (38.65%)
		21	58 (28.02%)
		22	25 (12.08%)
		23	4 (1.93%)
2	Gender	Female	145 (70.05%)
		Male	62 (29.95%)
3	Native	City	86 (41.55%)
		Town	70 (33.82%)
		Village	51 (24.64%)
4	Type of school for high school education	Private	167 (80.68%)
		Government	40 (19.32%)

The emotional intelligence (EI) scores (minimum possible score 0 and maximum possible score 160) of the medical students ranged from 55 to 148, with a mean score and standard deviation of 107.58 and 16.44 respectively. The distribution of scores on the four EI domains has been depicted in Table 2.

**Table 2:** Distribution of scores in the four EI domains

S.No	Emotional intelligence domain	Minimum score	Maximum score	Mean score	Standard deviation
1	Emotional Awareness (Max 40)	13	36	25.7391	4.7090
2	Emotional Management (Max 40)	0	38	25.1063	5.5755
3	Social Emotional Awareness (Max 40)	8	40	28.5700	5.2790
4	Relationship Management (Max 40)	8	40	28.1691	5.9834
5	Total Emotional Intelligence (Max 160)	55	148	107.58	16.4476

The association between age, gender, nativity and type of school with the scores in the overall EI are depicted in Table 3. It was observed that age, gender and nativity (urban/rural) had no significant influence on the EI scores. Students who went to government schools for their high school education had a significantly greater score on EI compared to students from private schools (*p* value = 0.032). This association remained statistically significant even after adjusting for other variables.

**Table 3:** Association between demographic factors and scores in the EI scale

S.No	Characteristics	Category	Below average EI	Above average EI	Chi Square *P* value	Adjusted *P* value
1	Age (years)	18	3 (75.0%)	1 (25.0%)	0.202	0.295
		19	16 (44.4%)	20 (55.6%)		
		20	48 (60.0%)	32 (40.0%)		
		21	26 (44.8%)	32 (55.2%)		
		22	10 (40.0%)	15 (60.0%)		
		23	1 (25.0%)	3 (75.0%)		
2	Gender	Female	72 (49.7%)	73 (50.3%)	0.796	0.913
		Male	32 (51.6%)	30 (48.4%)		
3	Native	City	41 (47.7%)	45 (52.3%)	0.549	0.242
		Town	34 (48.6%)	36 (51.4%)		
		Village	29 (56.9%)	22 (43.1%)		
4	Type of School for high school education	Private	90 (53.9%)	77 (46.1%)	0.032*	0.044*
		Government	14 (35.0%)	26 (65.0%)		

*Statistically significant *p* < 0.05

Logistic regression analysis of factors influencing response to emotional clinical vignettes is shown in Table 4. Age, nativity and type of school had no significant influence on the response to emotional clinical vignettes. Girls had a better response to the emotional situations in the vignettes than boys and this association tends towards statistical significance (adjusted *p* value = 0.056).

**Table 4:** Factors influencing response to the emotional clinical vignettes

S.No	Characteristics	Category	Below average response to emotional clinical vignettes	Above average response to emotional clinical vignettes	Chi square *P* value	Adjusted *P* value
1	Age (years)	18	4 (100.0%)	0 (0.0%)	0.346	0.843
		19	18 (50.0%)	18 (50.0%)		
		20	41 (51.3%)	39 (48.8%)		
		21	32 (55.2%)	26 (44.8%)		
		22	15 (60.0%)	10 (40.0%)		
		23	1 (25.0%)	3 (75.0%)		
2	Gender	Female	72 (49.7%)	73 (50.3%)	0.080	0.056^#^
		Male	39 (62.9%)	23 (37.1%)		
3	Native	City	51 (59.3%)	35 (40.7%)	0.372	0.253
		Town	34 (48.6%)	36 (51.4%)		
		Village	26 (51.0%)	25 (49.0%)		
4	Type of School for high school education	Private	89 (53.3%)	78 (46.7%)	0.846	0.453
		Government	22 (55.0%)	18 (45.0%)		
5	Total EI	Below average	58 (55.8%)	46 (44.2%)	0.534	0.452
		Above average	53 (51.5%)	50 (48.5%)		

^#^Tending towards statistical significance

### Qualitative study

**Fig. 1:**
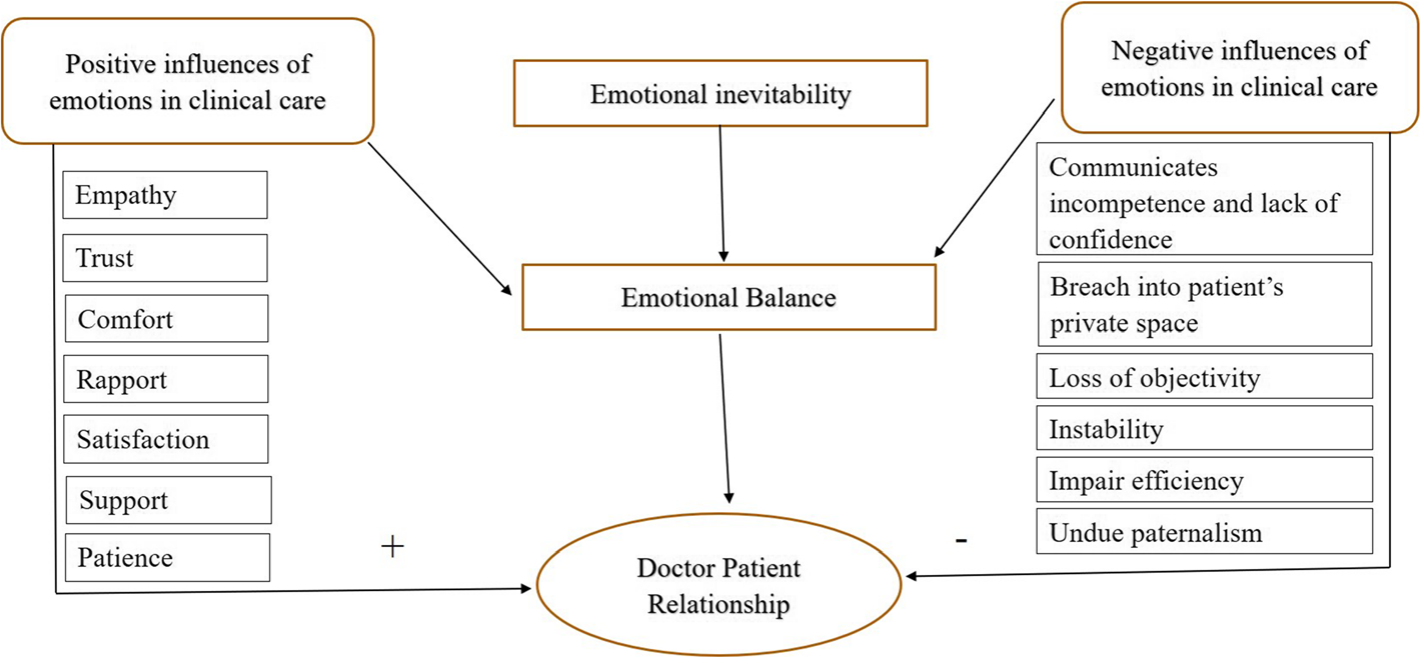
This figure shows the conceptual map of the various themes that emerged from the fish-bowl discussion on role of emotions in clinical medicine

The fishbowl discussion focussed on broad classification of three key themes with respect to medical students’ perceptions about emotions in the practice of medicine namely, (1) positive influences of emotions in clinical care, (2) negative influences of emotions, and (3) inevitability of emotions in the practice of medicine and the need for balancing emotions in medical practice. In the following paragraphs we describe the themes that emerged in discussion. Elaborate thematic analysis, along with representative quotes are presented as Additional file 2. The conceptual model that emerged from the discussion is shown in Fig. 1.

### Positive influences of emotions in clinical care

The students identified several positive influences of physician’s emotional intelligence in the practice of medicine. They felt that emotional intelligence helps the doctor establish a rapport with the patients and make them feel comfortable. It also helps the doctor provide the much-required emotional support. Emotional support is also an expression of empathy or relating to a patient as one’s own loved ones. This was expressed by a student,

*“the moment we understand the patient’s emotions and express our genuine emotions to the patients, we can care for the patient as we would care for a close relative. I think it is the best form of care” – female student, high schooling in government school.*

Empathy helps the doctor understand and treat the patient appropriately. While highlighting the importance of empathy, one of the students mentioned,

*“…the other day I saw a patient in the male medical ward. He was admitted for dressing of his diabetes foot ulcers. His blood sugar was poorly controlled. The physicians had refused to discharge him because his sugars were poorly controlled. He was refusing to stay back in the ward and there was a minor tiff between the physician and the patient. Later that evening, I went and spoke to this elderly man. I was genuinely empathetic about his situation and it must have reflected in the way I spoke to him. He opened up to me and narrated that his family was dependent on his painting work for their livelihood and he could not afford to stay in hospital much longer. I felt that the physicians could have had a more empathetic discussion with him on an emotional plane and avoided the tiff” – male student, high schooling in government school.*

Emotional intelligence also helps the doctor establish a trusting relationship and elicit satisfaction in the doctor-patient interaction. One of the students mentioned,

*“the patients expect a doctor who can understand their emotions and can show appropriate response to their emotions. What the patients expect is very important. It helps in establishing good doctor-patient relationships. If the patient has recently lost a loved one, and the doctor acknowledges the emotional distress caused by this, the doctor can connect immediately with the patient and can elicit full satisfaction in the encounter despite the grief situation” – female student, high schooling in government school.*

### Negative influences of emotions in clinical care

Alongside these positive influences, some of the students also highlighted the disadvantages of a doctor being emotionally involved with a patient. They felt that being emotionally involved with a patient can communicate lack of confidence and may lead to loss of objectivity. They also felt that it could lead to loss of efficiency of clinical care. In one narrative the student said,

*“…the aim of a doctor is to objectively assess a patient and treat the disease. It is not correct to lose the objectivity and become emotional” – female student, high schooling in government school.*

They also felt that it could lead to undue paternalistic attitudes towards the patient, which may hamper their engagement with their own care. One of the important emotions that a doctor experiences in a clinical encounter is anger. In case a patient does not adhere to treatment instructions, it can increase the doctor’s anger. In such circumstances, expression of anger can lead to undue paternalism and lack of sensitivity to the patient’s situation.

*“…sometimes it is important to get angry with a patient. If they are not listening to our instructions and are practicing unhealthy behaviours the anger will be useful to control their behaviours” – female student, high schooling in private school.*

It may also be unnecessary breach into the private emotional space of a patient. One of the students highlighted that,

*“…as a doctor, if I enter into the private emotional space of a patient it is crossing a professional line. It is not appropriate. Patients should be allowed to cry or have their emotional breakdown, but we should not enter their space” – male student, high schooling in private school.*

### Inevitability of emotions in the practice of medicine

As the fishbowl discussion grew to a close, the participants felt that feeling the emotions of patients and engaging with patients emotionally is inevitable. One of the participants said,

*“as human beings it is not possible to distance ourselves completely from the emotions associated with a patient or the suffering of a patient. Especially when it comes to new born babies, it is very difficult to stay free of emotions. It is important to acknowledge that we are emotional people” – female student, high schooling in government school.*

Another student in the group came forward with a point that emotions are inevitable, and doctors should figure out ways in which to express and handle the emotions. She said,

*“all doctors are humans too. We have to learn to have appropriate outlets for our emotions. If we try and bottle up our emotions, it will severely damage the mental health of the doctor. It can also lead to burn out. It is common that doctors working in intensive care units and oncology units suffer from depression and burn out.” – female student, high schooling in private school.*

After the discussions went on for about an hour and 10 min, it was time to summarize and conclude the session. During this time the facilitator stated all the important nuances of the discussion. The facilitator highlighted the interrelationship between the various facts that emerged in the discussion. The participants agreed that the themes described by the facilitator represented their discussion appropriately.

## Discussion

This mixed methods study initially quantified the emotional intelligence of the medical students. It was found that the students had emotional intelligence above the average, i.e., 40 being the maximum possible score, 20 is the average and the participants scored above 20 in each of the domains in the scale. Further it was found that students from government schools had greater emotional intelligence than those from private schools. Girls had better response to emotional clinical vignettes than boys, however this was not statistically significant. The qualitative follow up study to explore the status of emotional intelligence revealed that the students perceived several positive and negative influences of emotions in the practice of medicine. They agreed that emotions are inevitable, and a doctor should know to balance and actively engage with them.

### Emotional intelligence of government versus private school educated students

Our study observed the emotional intelligence of medical students who have done their high schooling in government public schools to be better than those who studied in private schools. This is in contradiction to the findings from other studies where private school students have outperformed government school students in emotional maturity and intelligence [15]. Students from rural areas and government schools have high degree of adaptability and ability to work as teams owing to lesser accessibility to resources compared to students from private schools. Government school students tend to be more aware of their emotions which are attributed to their social and communal upbringing during schooling. Private school students are probably brought up with an individualistic attitude with a focus on excellence of the individual. This difference in the school training has been reported in a previous study from the United States, which identified the distinction between individualistic and collectivistic trainings in schools [16]. The direct implication of this finding is that students who are exposed to resource limited practice settings and to the way of living, thinking and working in communities, tend to be more emotionally intelligent. Therefore, it may be possible to provide this kind of a communitarian environment that fosters emotional intelligence through community oriented medical education.

### Community oriented medical education to build emotional intelligence

Community oriented medical education (COME) is followed as an effective strategy worldwide to create socially conscious doctors [17]. “The aim of COME is to produce community oriented doctors who are able and willing to serve their communities and deal effectively with health problems at the primary, secondary and tertiary level.” [18] Undergraduate medical students are provided with clinical exposure in underserved or rural areas. This helps instil the sense of social responsiveness and motivation to students. For instance, the Christian Medical College in Vellore (CMC), India has addressed this need in its training programs by devising community-oriented programs for all medical graduates. CMC recognizes that relevant medical education and high-quality training are vital for students in a developing nation like India. Most of the students getting trained in medical colleges hail from middle and upper strata of the society and are unaware of the needs of rural community and urban slums. The hospital environment, where the classroom teaching takes place, is also not representative of the actual challenges faced in a community setting. To overcome this, the CMC took an initiative and established the Community and Health Development (CHAD), the main objective of which was to provide training in community based health care to medical students [18]. Such community oriented education can help the private school and urban students to develop a closer understanding of the lives of people living in rural areas and in under-resourced settings. As seen previously, this exposure may also help hone emotional intelligence among the students by exposing them to communitarian lives.

### Gender and emotional intelligence

Most of the tests of emotional intelligence suggest that women are on an average better than men at some forms of empathy, and men do better than women when it comes to managing distressing emotions [6, 7, 19]. The overall emotional intelligence obtained from the EI scale, of men and women were found to be almost equal from our study. This means that both men and women are capable of understanding, recognizing and managing their own emotions and that of others. This finding challenges the traditional notion that women score better than men in EI [20]. Another important question that arises in relation to gender and emotional intelligence is whether a greater EI among women translates to better communication skills, and better clinical care [21]. This study went a step ahead and assessed whether there is a difference in the way men and women respond to emotional clinical vignettes presented to them. It was found that, women performed better in responding to these vignettes compared to men. The implication of this finding is that women may be better at translating their EI into clinical care delivery compared to men. Medical education should focus on innovative strategies to teach male medical students to better express their emotions and translate their EI into clinical care. This can be achieved by cognitive reflection exercises periodically using emotional clinical vignettes and discussions.

### Medical students perceived emotional intelligence to be important

The qualitative follow up study clearly demonstrated that there was a wide spectrum in the opinion about emotions in the practice of medicine ranging from ‘emotions are not helpful’ all the way to ‘emotions are very important’. Fish bowl technique has been effectively used for cognitive reflection in several educational settings. In a medical education setting a previous study reported the simplicity and ease of use of this technique to teach clinical interviewing skills [22]. Another researcher described the use of fishbowl to impart knowledge on drug dependence in a pharmacology seminar [23]. To our best knowledge this is the first attempt at using the process to engage medical students in thinking about the role of emotions in the practice of medicine. Even though some students felt that emotions can hamper effective clinical care by coming in the way of efficient functioning of the doctors, the dominant narrative of the discussion was not denial of emotions but of how much of it to express and how much to control. The students finally did conclude that doctors, as humans, have to face emotional circumstances in clinical care and have to handle the emotions. This is a good starting point for actively engaging the students in emotional intelligence training. Engaging with the medical students with emotional intelligence training can start with such a fish-bowl or other cognitive reflection exercise. When the students are primed to think about the importance of emotional intelligence, they can be engaged in an ongoing curriculum for training in emotional intelligence.

### Holistic medical education requires emotional intelligence training

It is increasingly realized that a complete and holistic medical education cannot be devoid of emphasis on soft skills such as communication, empathy, ethics and emotional intelligence. Medical professionalism comprises of three essential components namely, ethical value system, knowledge and technical skills and interpersonal skills for working with patients and the medical care team [24]. Therefore, creating a professional medical doctor is a dynamic process of imparting not just knowledge and technical skills but also ethics and interpersonal skills. Emotional intelligence abilities are the building blocks that will allow medical college students to build healthy doctor patient relationships in the future. There has been very little focus globally on training medical students in emotional intelligence. Therefore, medical educators must include emotional intelligence testing and training in the syllabus.

This study emphasizes the need for integration of EI training into undergraduate medical education. Several attempts have been made by scholars to attempt to teach EI to medical students in training [25]. Cognitive reflection exercises, practical role play scenarios, socio-drama techniques and conflict resolution methods have been adopted to train medical students to understand and perceive their own emotions [26]. It has also been proposed that systematic reflection of a student’s emotions in an individual, face to face discussion with a mentor about emotionally trying clinical situations can be the starting point in emotional intelligence training.

## Conclusion

In conclusion, this study showed that the levels of emotional intelligence were good among men and women in the college that was studied. Communitarian education settings can hone emotional intelligence skills, thus emphasizing the need for community oriented medical education. It also showed that though men and women have similar EI, women are better at responding appropriately to emotional clinical vignettes, thus indicating ability to effectively translate EI to better clinical care. Finally, the study also showed that effective cognitive reflection exercises can help students understand the importance of emotions in the practice of medicine and this can be a good starting point for emotional intelligence skill building.

## Additional files

Additional file 1: Emotional intelligence among medical college students, questionnaire. This file provides the questionnaire used to assess the emotional intelligence of the medical college students. (DOCX 19 kb)

Additional file 2: Detailed analysis of the themes that emerged from the fishbowl discussion on role of emotions in clinical care. This file provides the themes that emerged from the analysis of the fishbowl discussion along with verbatim quotes. (DOCX 18 kb)

## Abbreviations

ATCOM: Attitudes and communication
CHAD: Community health and development
CMC: Christian medical college
COME: Community oriented medical education
EI: Emotional intelligence
