# Supplementary material for: Emotional intelligence among medical students: a mixed methods study from Chennai, India
Source: BMC Med Educ. 2018 May 4;18:97. doi: 10.1186/s12909-018-1213-3 (PMC5935999; doi:10.1186/s12909-018-1213-3)
Supplement: Supplementary file 1 — Emotional intelligence among medical college students, questionnaire. This file provides the questionnaire used to assess the emotional intelligence of the medical college students. (DOCX 19 kb) [file 12909_2018_1213_MOESM1_ESM.docx]

EMOTIONAL INTELLIGENCE AMONG MEDICAL COLLEGE STUDENTS - Questionnaire

Year of Study : ( II, III, Final Year)

Name :

Roll no. :

AGE :______________ GENDER : M / F

NATIVE : City / Town / Village TYPE OF SCHOOL : Private / State

TASK-1

QUICK EMOTIONAL INTELLIGENCE TEST

Rank each statement as follows:

0 (Never) 1 (Rarely) 2 (Sometimes) 3 (Often) 4 (Always)

Emotional Awareness - TOTAL: _____________

| 0 1 2 3 4 | My feelings are clear to me at any given moment |
| --- | --- |
| 0 1 2 3 4 | Emotions play an important part in my life |
| 0 1 2 3 4 | My moods impact the people around me |
| 0 1 2 3 4 | I find it easy to put words to my feelings |
| 0 1 2 3 4 | My moods are easily affected by external events |
| 0 1 2 3 4 | I can easily sense when I am going to be angry |
| 0 1 2 3 4 | I readily tell others my true feelings |
| 0 1 2 3 4 | I find it easy to describe my feelings |
| 0 1 2 3 4 | Even when I’m upset, I’m aware of what is happening to me |
| 0 1 2 3 4 | I am able to stand apart from my thoughts and feelings and examine them |

Emotional Management - TOTAL: ___________

| 0 1 2 3 4 | I accept responsibility for my reactions |
| --- | --- |
| 0 1 2 3 4 | I find it easy to make goals and stick to them |
| 0 1 2 3 4 | I am an emotionally balanced person |
| 0 1 2 3 4 | I am a very patient person |
| 0 1 2 3 4 | I can accept critical comments from others without becoming angry |
| 0 1 2 3 4 | I maintain my composure, even during stressful times |
| 0 1 2 3 4 | If an issue does not affect me directly, I don’t let it bother me |
| 0 1 2 3 4 | I can restrain myself when I feel angry towards someone |
| 0 1 2 3 4 | I control urges to overindulge in things that could damage my well being |
| 0 1 2 3 4 | I direct my energy into creative work or hobbies |

Social Emotional Awareness - TOTAL: ___________

| 0 1 2 3 4 | I consider the impact of my decisions on the other people |
| --- | --- |
| 0 1 2 3 4 | I can easily tell if the people around me are becoming annoyed |
| 0 1 2 3 4 | I sense it when a person’s mood changes |
| 0 1 2 3 4 | I am able to be supportive when giving bad news to others |
| 0 1 2 3 4 | I am generally able to understand the way other people feel |
| 0 1 2 3 4 | My friends can tell me intimate things about themselves |
| 0 1 2 3 4 | It genuinely bothers me to see other people suffer |
| 0 1 2 3 4 | I usually know when to speak and when to be silent |
| 0 1 2 3 4 | I care what happens to other people |
| 0 1 2 3 4 | I understand when people’s plans change |

Relationship Management - TOTAL: ___________

| 0 1 2 3 4 | I am able to show affection |
| --- | --- |
| 0 1 2 3 4 | My relationships are safe places for me |
| 0 1 2 3 4 | I find it easy to share my deep feelings with others |
| 0 1 2 3 4 | I am good at motivating others |
| 0 1 2 3 4 | I am fairly cheerful person |
| 0 1 2 3 4 | It is easy for me to make friends |
| 0 1 2 3 4 | People tell me I’m sociable and fun |
| 0 1 2 3 4 | I like helping people |
| 0 1 2 3 4 | Others can depend on me |
| 0 1 2 3 4 | I am able to talk to someone when they are very upset |

TASK-2

EMOTIONAL INTELLIGENCE CLINCAL VIGNETTES

Please read the following scenarios and respond by marking/circling any one of the given answers for the question asked.

(1) You are an intern working in the trauma and casualty ward. There is a sudden rush of 18 patients into the casualty, when their van toppled at the turn of the road. One of them has sustained a severe head injury and is unconscious. The other 17 have sustained various degrees of fractures, cuts, lacerations, bruises and injuries. You call your friends from the surgery wards to help and start attending to the unconscious patient. Meanwhile, one of the other patients, who has a minor injury over his left hand is angry and restless and shouts at you. He calls you and says, “I don’t know why they keep such young and ineffective doctors in the casualty. Go run and get your seniors. I want this wound to be attended to immediately”.

How would you respond to this situation?

1. Leave the scene and run to call the PG or the Assistant Professor on call that day
2. Call the security to control the angry patient and continue your work on the unconscious patient.
3. Shout back firmly, “Who do you think you are? Shut up and lie down in that bed. Don’t you see I am attending to an unconscious patient? If you don’t shut up now, I will throw you out”
4. Put your hand on the angry patient’s shoulder, try to calm him down and speak gently but firmly, “please understand that I am attending to a serious patient. When I get free I will definitely take care of you or I will call somebody else to take care of you. Please be patient”

(2) You are sitting in the Hypertension clinic. Every month an elderly man, Mr.Sadasivam, comes to your clinic, specifically asks for you and sees you. You regularly check his BP and write his prescription. Last week you heard from the local health worker that Mr.Sadasivam’s son met with a motorcycle accident in the highway and died on the spot. Today, Mr.Sadasivam walked into the clinic and as soon as he sat down in front of you he held your hand and started crying uncontrollably. His body was shaking and he was inconsolable.

What would you do?

a. Get up and walk out, allowing him to cry in private.

b. Put your hand on his shoulder, console him and say “don’t cry, please don’t cry”.

c. Put your hand on his shoulder, and stay silent ,allowing him to cry.

d. Put your hands on his shoulder and say, “did your son wear helmet? If he had worn helmet he would have been alive now”.

(3) Once in every 3 days an elderly man holding a walking stick comes into your clinic with complaints of severe back pain, knee pain and shoulder pain. He is neither diabetic nor hypertensive and lives alone in his own hut in the nearby village. He takes a bus, spends 7 rupees for the ticket and comes specifically to meet you once in three days. He always asks for diclofenac injection. You just gave him an injection last week and he has come back today with a request for the injection. You are concerned about repeated injections. The patient is insisting that he wants only injection.

What will you do?

a. Refuse the injection firmly and give him a prescription for tablets

b. Tell him (angrily), “If you keep asking me for injections, I will not see you anymore”.

c. Give him a placebo injection (neurobion)

d. Ignore his demand and talk about something else and give him tablets.

(4) You are treating an elderly lady for congestive heart failure. She lives alone. Her children visit her once a month and give her some money to manage her monthly expenses. She cooks and eats on her own and is very lonely. She goes into depression most of the times and cries often. When she visits you this time she asks you whether she can go and meet the faith healer who is an expert in removing the evil curse of spirits. She believes that her family enemies have done the black magic on her which is the main reason for worsening of her symptoms. She says her son will come and take her tomorrow to the healer and drop her back after 3 days. She will stay those 3 days in the town at her son’s house. She asks you whether she can travel that far (45 km) in the bus with her son.

How will you respond to this request?

a. Allow her to go to her son’s place

b. Advise her that faith healing and black magic are all superstitious and she has to continue taking treatment from you

c. Tell her that her symptoms will worsen if she goes to the faith healer and so she must not go.

d. Call her son’s phone number and tell him that the trip to faith healer is a bad idea.
