# Supplementary material for: Emotional intelligence among medical students: a mixed methods study from Chennai, India
Source: BMC Med Educ. 2018 May 4;18:97. doi: 10.1186/s12909-018-1213-3 (PMC5935999; doi:10.1186/s12909-018-1213-3)
Supplement: Supplementary file 2 — Detailed analysis of the themes that emerged from the fishbowl discussion on role of emotions in clinical care. This file provides the themes that emerged from the analysis of the fishbowl discussion along with verbatim quotes. (DOCX 18 kb) [file 12909_2018_1213_MOESM2_ESM.docx]

**Additional File 2**

**Detailed analysis of the themes that emerged from the fishbowl discussion on role of emotions in clinical care**

**Positive influences of emotions in clinical care:**

The students highlighted several positive influences of emotions in clinical care.

*Rapport:*

Often the clinical encounter is a situation where the doctor and patient are coming together for the first time. Many times it may be the only encounter. In these circumstances establishing rapport quickly is very important. Emotions are very important in establishing rapport with a patient. One of the discussants mentioned,

*“…connecting emotionally with a patient helps us quickly establish rapport and elicit a better history and understand the problems of the patient well” – male student*

An emotionally engaged doctor elicits a far better history compared to a distant and aloof doctor. Apart from eliciting a good history, a good rapport also ensures that instructions are adhered to and treatment is followed diligently.

*Trust:*

Trust is the fundamental basis of a good doctor-patient relationship. The patients are in a state of vulnerability and therefore they need reassurances that the doctor is trustworthy. One of the important methods of communicating trustworthiness is understanding and relating to the patients’ emotions. A student said,

*“…emotions are the basis of good doctor patient relationship. only if we show emotion we can win the trust of the patient”- male student*

On the other hand, another participant also worried that expressing excessive concern, worry and anxiety about treatment of a difficult condition in a patient, though natural, may hamper the level of trust the patient has on the doctor. Therefore, he mentioned that expression of emotions of the physician can lead to reduced trust.

*“…expressing some emotions like fear and anxiety can decrease the confidence and trust of the patients”- male student*

Expressing and responding to appropriate emotions may help in trust building, however, certain emotions may also hamper the levels of trust.

*Satisfaction:*

Satisfaction of patients’ expectations is essential for good doctor patient relationships and for continuation of care, especially in chronic diseases. One student mentioned,

*“the patients expect a doctor who can understand their emotions and can show appropriate response to their emotions. What the patients expect is very important. It helps in establishing good doctor-patient relationships. If the patient has recently lost a loved one, and the doctor acknowledges the emotional distress caused by this, the doctor can connect immediately with the patient and can elicit full satisfaction in the encounter despite the grief situation” – female student*

Trust and satisfaction are two sides of the clinical encounter. Emotional sensitivity of the physician can influence both.

*Empathy:*

Being sensitive to emotional experiences of patients and responding appropriately to these emotional experiences was mentioned as being very helpful in developing empathy. One of the participants narrated an instance where understanding the emotions of a patient helped him empathize with the patient and appreciate the need for empathy. He said,

*“…the other day I saw a patient in the male medical ward. He was admitted for dressing of his diabetes foot ulcers. His blood sugar was poorly controlled. The physicians had refused to discharge him because his sugars were poorly controlled. He was refusing to stay back in the ward and there was a minor tiff between the physician and the patient. Later that evening, I went and spoke to this elderly man. I was genuinely empathetic about his situation and it must have reflected in the way I spoke to him. He opened up to me and narrated that his family was dependent on his painting work for their livelihood and he could not afford to stay in hospital much longer. I felt that the physicians could have had a more empathetic discussion with him on an emotional plane and avoided the tiff” – male student*

Relating to the emotions of a patient helps in developing empathy and thus creates a strong doctor patient relationship.

*Comfort:*

Comfort is a state of ease, freedom from pain and mental distress. Illness is a very stressful state. In this state, comfort is essential. A student mentioned in the discussion, the importance of understanding the emotional distress of a patient and comforting her,

*“…when a patient is alone in the operation theatre with a lot of strange people around her, all she expects is a comforting doctor who understands her fear and anxiety and comforts her” – male student*

Such a comforting gesture can be priceless in high stress circumstances like an operation theatre or emergency room.

*Support:*

Support is the perception that the patient is actually cared for during the time of illness. It can be physical, emotional, informational or appraisal support. Emotional support is the offering of empathy, concern, affection, love, care, motivation and encouragement in the process of treatment and healing. An enthusiastic discussant mentioned,

*“only if we allow ourselves (doctors) to experience the emotions of a patient, we can understand their need for social support. We can provide emotional support which can help in faster healing” – female student*

Emotional support is essential for the process of healing of a patient as well as for the patient attenders. Emotional support is also an expression of empathy or relating to a patient as one’s own loved ones. This was expressed by a student,

*“the moment we understand the patient’s emotions and express our genuine emotions to the patients, we can care for the patient as we would care for a close relative. I think it is the best form of care” – female student*

*Patience:*

One of the participants mentioned that emotional sensitivity helps her understand the need for patience and tolerance.

*“being sensitive to emotions teaches me patience. It makes me more tolerant to the anger that the patient or the patient’s attender expresses. It helps me put myself in the shoes of the patient and understand the emotional circumstances” – female student*

**Negative influences of emotions in clinical care:**

During the discussion each time there were presentations of positive influences of emotions in the clinical encounter, there were interesting counter arguments which highlighted the negative influences.

*Being emotional communicates incompetence and lack of confidence:*

One particular student mentioned vehemently that being emotional can express weakness and lack of confidence to the patient. Rather than being the strong pillar of strength that the patient is expecting from the doctor, the doctor turns out to be a weak and emotional person, which may be counterproductive to the clinical outcome.

*“being emotional is being weak. Doctors have to be pillars of strength for the patients. They cannot themselves be emotionally affected by the patient’s condition.” – male student*

When the student mentioned this, there was immediately a lot of small discussions within the large group and there seemed to be several voices agreeing and several disagreeing with this view.

*Breach into the patient’s private space:*

In increasingly individualistic societies where personal and professional boundaries are clearly demarked, entering the emotional space of a patient may be considered as a breach of privacy. Another remark made by a student which received a lot of discussions was,

*“…as a doctor, if I enter into the private emotional space of a patient it is crossing a professional line. It is not appropriate. Patients should be allowed to cry or have their emotional breakdown, but we should not enter their space” – male student*

How much of entry into personal lives of patients is acceptable and what is the boundary seems to be a matter of important consideration. Further it is also important to understand whether such emotional engagement with patients is possible without breaching their privacy to a great extent.

*Loss of objectivity:*

Two participants in the discussion made interesting remarks which related to loss of objectivity when the doctor gets emotionally involved with a patient. In one narrative the student said,

*“…the aim of a doctor is to objectively assess a patient and treat the disease. It is not correct to lose the objectivity and become emotional” – female student*

Being highly sensitive to the emotions of a patient and experiencing their emotions, can lead to loss of objectivity. She was afraid that relating to the pain that will be experienced by a patient due to a procedure, may prevent the doctor from performing the procedure. In yet another narrative of the same theme, another student explained how treating her own father severely impaired her objectivity and her performance because of emotional involvement.

*“…last week my father was unwell. I had to make a decision whether to start an intravenous line and infuse intravenous fluids at home as he was dehydrated. But because he is my father and because of my emotional connection with him, I couldn’t make the decision. This is what emotions can do to performance of a doctor” – female student*

*Instability:*

A doctor who makes decisions out of emotional considerations can be quite unstable in the decision making process. One of the participants remarked that,

*“an emotional person is often seen as an unstable person” – male student*

This again led to a lot of discussions on whether an emotional person can be trusted to make reasonable decisions.

*Impaired efficiency:*

The same student who made the much discussed statement that being emotional is a sign of weakness, requested a re-entry to the central discussion and made yet another interesting and much discussed statement,

*“…it is not enough if the doctor speaks kind words and shows empathy. Being a doctor requires a lot of skill and demands performance under high pressure. Holding the patient’s hands in the operation theatre and uttering kind words is not going to save the life. Operating efficiently under high stress might actually save the life. Therefore, emotions have no place, especially in high stress professions like neurosurgery”- male student*

He argued that being emotional will impair the efficiency of the surgeon in the operation room. While there are people in the operation room who can take care of comforting and caring for the patient, it cannot be the responsibility of the surgeon who should not divert his/her attention from his/her performance which is of utmost importance.

*Undue paternalism:*

One of the important emotions that a doctor experiences in a clinical encounter is anger. In case a patient does not adhere to treatment instructions, it can increase the doctor’s anger. In such circumstances, expression of anger can lead to undue paternalism and lack of sensitivity to the patient’s situation.

*“…sometimes it is important to get angry with a patient. If they are not listening to our instructions and are practicing unhealthy behaviours the anger will be useful to control their behaviours” – female student*

Having discussed both the positive and negative aspects of emotions in a clinical encounter, the students did stand divided in their opinions. However, towards the latter part of the fishbowl discussion, a new theme of discussion started emerging, which emphasized on the inevitability of emotions in the process of providing clinical care.

**Emotional inevitability**

Two discussants, supported by a small group of students who were in the outer circle emerged with a theme of inevitability of perceiving emotions and emotional experiences in the process of providing clinical care. They joined the central discussion and introduced this theme. One of them said,

*“as human beings it is not possible to distance ourselves completely from the emotions associated with a patient or the suffering of a patient. Especially when it comes to new born babies, it is very difficult to stay free of emotions. It is important to acknowledge that we are emotional people” – female student*

She reinforced that all humans are subject to various emotions and doctors are no different. While providing care for patients, it is impossible for doctors to separate themselves from the emotions associated with the suffering or the treatment. Another participant gave this a stronger tone when he said,

*“…doctors are not robots. We are people with emotions. That is what differentiates us from robots. Therefore, we should have emotions” – male student*

One of the female students in the group came forward with a point that emotions are inevitable, and doctors should figure out ways in which to express and handle the emotions. She said,

*“all doctors are humans too. We have to learn to have appropriate outlets for our emotions. If we try and bottle up our emotions, it will severely damage the mental health of the doctor. It can also lead to burn out. It is common that doctors working in intensive care units and oncology units suffer from depression and burn out.” – female student*
